# Factors Affecting the Distribution Pattern of Wild Plants with Extremely Small Populations in Hainan Island, China

**DOI:** 10.1371/journal.pone.0097751

**Published:** 2014-05-15

**Authors:** Yukai Chen, Xiaobo Yang, Qi Yang, Donghai Li, Wenxing Long, Wenqi Luo

**Affiliations:** Key Laboratory of Protection and Development Utilization of Tropical Crop Germplasm Resources, Ministry of Education, College of Horticulture and Landscapes, Hainan University, Haikou 570228, Hainan, China; Wuhan Botanical Garden, Chinese Academy of Sciences, Wuhan, China, China

## Abstract

Understanding which factors affect the distribution pattern of extremely small populations is essential to the protection and propagation of rare and endangered plant species. In this study, we established 108 plots covering the entire Hainan Island, and measured the appearance frequency and species richness of plant species with extremely small populations, as well as the ecological environments and human disturbances during 2012–2013. We explored how the ecological environments and human activities affected the distribution pattern of these extremely small populations. Results showed that the extremely small populations underwent human disturbances and threats, and they were often found in fragmental habitats. The leading factors changing the appearance frequency of extremely small populations differed among plant species, and the direct factors making them susceptible to extinction were human disturbances. The peak richness of extremely small populations always occurred at the medium level across environmental gradients, and their species richness always decreased with increasing human disturbances. However, the appearance frequencies of three orchid species increased with the increasing human disturbances. Our study thus indicate that knowledge on how the external factors, such as the ecological environment, land use type, roads, human activity, etc., affect the distribution of the extremely small populations should be taken for the better protecting them in the future.

## Introduction

Plant population is the most basic level of plant ecology, connecting plant individuals with communities and ecosystems. The ecological patterns of a plant population are characterized by its population dynamics, as well as the relationships between the population and the external environments [1]. Many studies of common species and their companion species have been previously conducted, and focused on the population structure, life history, and the relationship between the population and environment, etc [2]–[4]. But researches concerning the mechanism of endangerment or the threat on the rare and endangered plants are seldom studied [5]–[8]. These researches, however, become important to biodiversity protection across the world.

Both internal and external factors cause plants to become vulnerable to extinction. Internal factors refer to the plant biological characteristics, including failures in heritability, reproduction, viability, and adaptability [9], [10]. External factors include both natural and human factors. Natural factors refer to the ecological environment, including climate, topography, soil, and other biological factors [11], [12]. For example, many rare and endangered plants are only distributed in narrow area with a special microclimate. Some of them occur only in temporary habitats, fragmented habitats or “ecological islands” [13], [14]. Human factors refer to human disturbances, roads, and land use types. Most of them have become the harmful factors impacting on rare and endangered plants [15]–[19].

Hainan is the largest island of the Indo Burma Biodiversity Hotspot, and has the best preserved and the most extensive tropical forests in China. It contains rich rare and endangered plant species, and therefore, becomes a priority for species conservation in China [20], [21]. But plants on Hainan Island often have a narrow distribution with few individuals, and thus easily become endangered and extinct because of geographical isolation of this Island from the mainland. Additionally, the introduction of alien plants, habitat destruction, and the overexploitation have accelerate the shrink and extinction of these rare and endangered plant populations [22], [23].

The State Forestry Administration of China has been carrying out the “Conservation Program for Wild Plants with Extremely Small Populations in China (from 2011 to 2015)”. In this plan, 24 wild plant species with extremely small population in Hainan are recorded. The term “extremely small population” refers to a population with a narrow geographical distribution that has been disturbed and constrained by external factors over a long time, and contains less individuals than the minimal number requirements inhibiting from extinction. Some of these plants are qualified as the biological resources important to the country's ecology, science, culture, and economics [24]. For the majority of extremely small populations in China, the main factors causing them susceptible to endangerment or extinction are habitat destruction, overexploitation, and environmental pollution [25]. Previous each study on the mechanism of endangerment and protection countermeasures for rare and endangered plants has only focused on a single species [6], [7], [12], leaving open the question of whether there are common mechanisms on the external factors affecting different endangered plants. In this study, we assessed the population sizes and conservation situations of several extremely small populations, studied the characteristics of their habitats, and explored the effect of ecological environment, roads, and human activity on the distribution patterns of these plants. We use our findings to highlight the knowledge gaps in the conservation strategies for these endangered species.

## Materials and Methods

### Study Area

**Figure 1:**
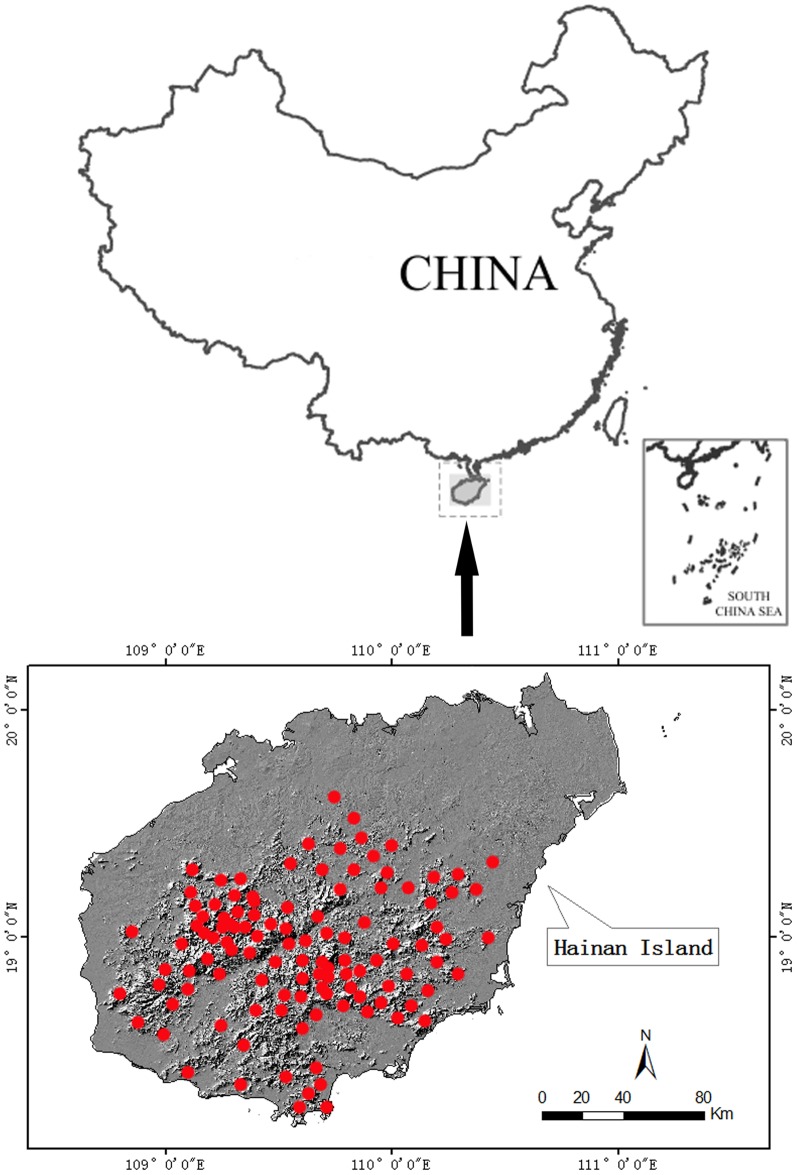
Hainan map and distribution of the 108 sample plots of the field survey. The red circles indicate plots.

This study was conducted in Hainan Island which is located at the southernmost tip of China (18°10′–20°10′ N, 108°37′–111°03′E) and covers a total area of 33,900 km^2^. The island is high at its center and relatively low around its perimeter. The highest peak is Wuzhishan Mountain, with an altitude of 1,867 m (Figure 1). Currently, the island's central and southern mountainous areas contain a rich biodiversity, with many rare and endangered wild plants. However, in the northern and coastal areas, these plant have been almost destroyed by rubber plantations and other forms of agriculture, as well as industrial and urban developments [26], [27]. Hainan has a wet tropical monsoon climate, with a distinct wet season from May to October and a dry season from November to April. The annual average temperature is 23°C–25°C, and the annual average rainfall is 1,500–2,500 mm [28].

### Field Survey

A total of 108 plots were established covering areas of tropical rainforest, tropical monsoon forest, mangrove forest, tropical coniferous forest, tropical evergreen and semi-evergreen shrubs, semi-natural vegetation, and artificial vegetation. Plots were mainly distributed in the central and southern mountainous areas because of their rich rare and endangered wild plants (Figure 1). Each plot included a 20×20 m-quadrat distributed at the central point, companied with four 20×20 m-quadrates distributed in the four diagonal directions from the central points.

A comprehensive survey was performed to identify plant species names, and measure the all individuals across the entirety of Hainan Island during 2012–2013. The survey of wild plants with extremely small populations in Hainan is permitted and approved by the Ministry of Agriculture of the People's Republic of China, and the Wildlife Protection Bureau of Hainan Province. Our work was conducted with a focus on the protection of wildlife and causing no threats or damage to wildlife, especially to endangered or protected species.

The list of the surveyed plants was in accordance with the species names of “Conservation Program for Wild Plants with Extremely Small Populations in China” [24], including *Cycas changjiangensis*, *Michelia odora*, *Chieniodendron hainanense*, *Ilex kaushue*, *Horsfieldia kingii*, *Paranephelium hainanense*, *Sonneratia hainanensis*, *Lumnitzera littorea*, *Hopea hainanensis*, *Bulbophyllum hainanense*, *Ceratostylis hainanensis*, *Cymbidium eburneum*, *Cymbidium insigne*, *Oxystophyllum changjiangense*, *Dendrobium hainanense*, *Dendrobium sinense*, *Dendrobium strongylanthum*, *Doritis pulcherrima*, *Pinalia quinquelamellosa*, *Dendrolirium tomentosum*, *Gastrochilus acinacifolius*, *Phaius hainanensis*, *Sunipia hainanensis*, and *Thrixspermum odoratum*. We found that four species (*Ilex kaushue*, *Bulbophyllum hainanense*, *Phaius hainanensis*, *Sunipia hainanensis*) that had previously been recorded in Hainan Island were not found in our field survey. Thus, further investigation is required to determine whether these species have disappeared from the wild. Among the remaining 20 species with extremely small populations, we recorded the distributed area, individual number and habitat characteristics (see Appendix S1).

### Distribution Patterns

Distribution pattern refers to the appearance frequency and species richness of the extremely small populations. The appearance frequency of each extremely small population was calculated as the ratio of the number of quadrates in which a plant was found to the five quadrates.

### Measurement of Affecting Factors

We measured 13 variables which were divided into five types. (1) Topographical: altitude, slope aspect, slope gradient, and exposure of rock. Altitude, slope aspect, and slope gradient were measured using the global positioning system, a compass, and a slope measuring instrument, respectively. And the exposure of rock was estimated by the ratio of the exposed surface stone area to the total area of the quadrates. (2) Climate: annual average temperature and average annual precipitation. These variables were extracted for each site from the WorldClim database (http://www.worldclim.org) with a spatial resolution of 0.0083° (approximately 1 km^2^ at the equator) [29]. (3) Vegetation: species richness and canopy structure. Species richness was calculated as the mean number of species found in the sample. Canopy structure included both leaf area index and canopy density, which were measured using hemispherical photographs at a height of 1.5 m. Leaf area index, referred to as *LAI* 4 Ring (*LAI*), is the effective leaf area index integrated over the zenith angles 0–60°. Canopy density is the percentage of shelter sky seen from beneath a forest canopy. A hemispherical photograph was taken in each 20×20 m-quadrat using a Nikon 4500 Coolpix Digital camera (Nikon Corporation, Tokyo, Japan) fitted with a Nikon FC-E8 fisheye converter (183° field-of-view, orthographic projection) [30]. The hemispherical images were analyzed with Gap Light Analyzer (GLA) software, Version 2.0 [31]. (4) Road: road quality and distance between population and road. According to the width and the lay material of road, road quality was assigned values of 1, 2, 3, and 4, which represent rural sandstone road (width 2–3 m), rural cement road (width 3–4 m), township road (width 4–5 m), and county road (width 5–6 m), respectively. Distances between population and road were measured using a tape measure and the global positioning system combined with Google Earth. (5) Human activity: surrounding population density and land use type. Surrounding population density was assigned values of 1, 2, 3, and 4, which indicated a depopulated zone, village, town, and suburb, respectively. Land use type was assigned values of 1, 2, 3, and 4, which represent dense forest, sparse woodland or shrubland, wasteland, and plantation or agricultural land, respectively.

### Data Analysis

In this study, principal components analysis (PCA) was first used to analyze the 13 variables to reduce the dimension of factors. The top eight variables with absolute eigenvalues more than 0.620 were selected. Then the Canonical correspondence analysis (CCA) and the bivariate correlation analysis were performed to examine the relationship of these selected variables with the appearance frequency of each extremely small population.

We compared the species richness of the extremely small populations that were found at the different levels of the each selected variables using one-way ANOVA.

We divided the altitude values into six levels from 0 to 1800 m: 0–300 m (8 samples), 300–600 m (22 samples), 600–900 m (16 samples), 900–1200 m (44 samples), 1200–1500 m (10 samples), and 1500–1800 m (8 samples), and then analyzed the species similarity between neighboring altitudes using the following Jaccard similarity coefficient index: C_j_ = C/(A+B−C) [32], where A and B represent the number of extremely small populations found in each neighboring altitude, and C indicates the number of common species found in each neighboring altitude.

We divided the slope aspect into eight ranges: north slope (0°–22.5° and 337.5°–360°, 16 samples), northeast slope (22.5°–67.5°, 16 samples), east slope (67.5°–112.5°, 14 samples), southeast slope (112.5°–157.5°, 11 samples), south slope (157.5°–202.5°, 12 samples), southwest slope (202.5°–247.5°, 8 samples), west slope (247.5°–292.5°, 12 samples), and northwest slope (292.5°–337.5°, 18 samples), and then calculated the frequency for each plant species with extremely small populations occurred at the eight slope aspects. Before the CCA analysis, the slope aspect was cosine transformed, and resulted in values ranged from 0 (south) to 2 (north) [33].

We divided the slope gradient into six levels ranged from 0° to 90°: 0°–15° (15 samples), 15°–30° (34 samples), 30°–45° (40 samples), 45°–60° (9 samples), 60°–75° (4 samples), and 75°–90° (6 samples). Canopy density was divided into six levels from 0.4 to 1.0: 0.4–0.5 (8 samples), 0.5–0.6 (7 samples), 0.6–0.7 (12 samples), 0.7–0.8 (28 samples), 0.8–0.9 (24 samples), and 0.9–1.0 (29 samples). Distance correlation analysis was then performed to calculate the Pearson similarity coefficient, using the presence/absence data of each extremely small population for the six levels of slope gradient or canopy density. Distance between population and road was divided into five levels: 0–200 m (25 samples), 200–400 m (26 samples), 400–600 m (21 samples), 600–800 m (16 samples), and 800–1000 m (20 samples). Road quality, surrounding population density, and land use type were each divided into four levels according to their 1–4 scales.

PCA, bivariate correlation analysis and distance correlation analysis were performed using SPSS 19.0. CCA was performed using CANOCO version 4.5 for Windows, and a Monte Carlo permutation test was conducted with 1000 randomizations for the significance of both the variables and the ordination axes [34]. Figures were created with SigmaPlot 12.0.

## Results

### PCA of Affecting Factors

PCA showed that the accumulative contribution rate of the three principal components were 58.380% (Table 1). Thus the top eight principal component with absolute eigenvalues more than 0.620 were found. They were road (−0.821), canopy density (0.812), altitude (0.721), slope gradient (0.716), surrounding population density (−0.707), slope aspect (0.646), road quality (−0.660), and land use type (0.622). These eight variables, therefore, are predicted to affect the distribution patterns of wild plants with extremely small populations.

**Table 1:** Principal component values, eigenvalues, and contribution rate of variables in principal components analysis.

Variable	Principal component		
	1	2	3
Species Richness	0.616	0.077	−0.070
Leaf area index	0.612	0.347	−0.231
Canopy Density	0.812	0.433	−0.083
Annual average temperature (°C)	−0.313	−0.168	−0.465
Average annual precipitation (mm)	0.432	−0.014	0.399
Road Quality	−0.660	0.245	0.224
Distance between Population and Road (m)	0.233	−0.821	0.121
Exposure of rock (%)	−0.591	−0.247	0.031
Altitude	0.721	−0.185	0.105
Slop Aspect	−0.126	0.646	0.386
Slop Gradient	−0.071	−0.041	0.716
Surrounding Population Density	−0.707	0.261	−0.028
Land use type	0.319	0.622	−0.403
Eigenvalue	3.294	1.717	1.395
of variance (%)	27.446	14.307	11.627
Cumulative (%)	27.446	41.753	58.380

### Relationships Between Appearance Frequency and Affecting Factors

**Figure 2:**
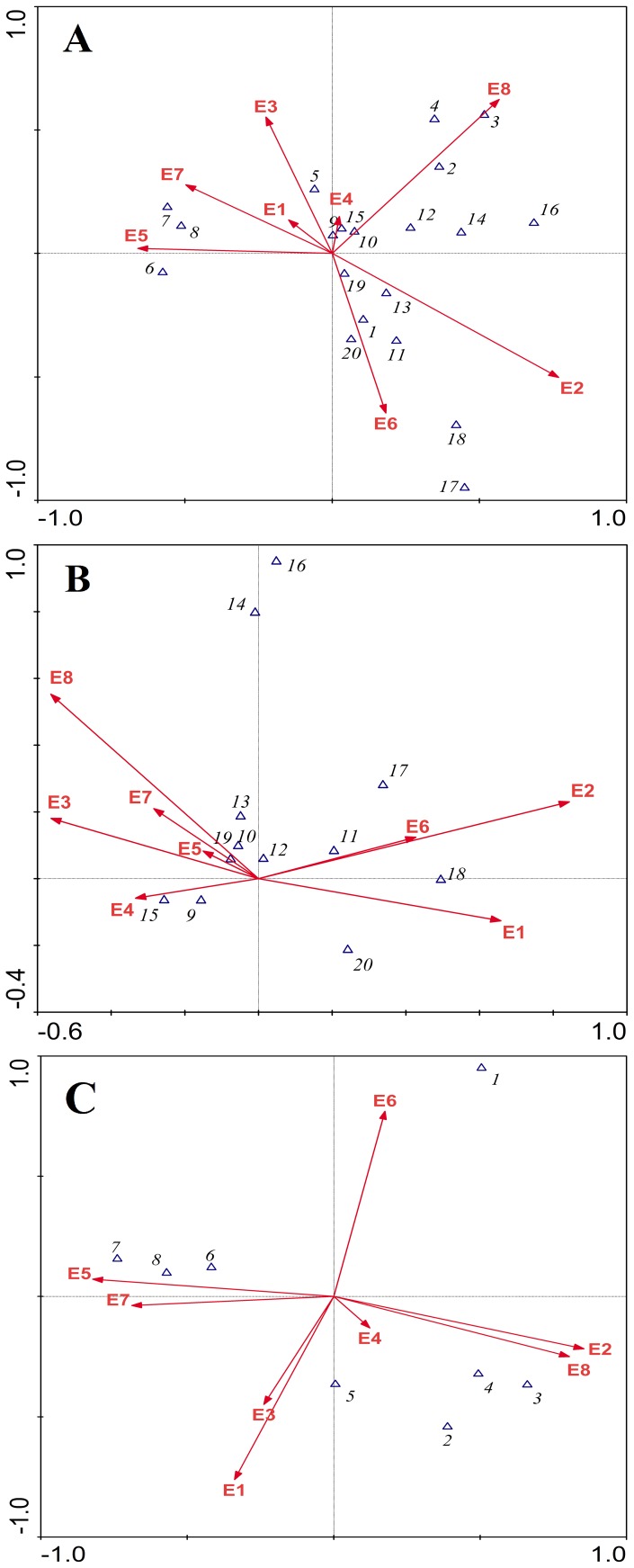
Canonical correspondence analysis (CCA) between (A), total species; (B), orchid species; (C), non-orchid species with extremely small populations and eight factors. The numbers from 1 to 20 represent the 20 species with extremely small populations, as shown in Appendix S1. E1, canopy density; E2, altitude; E3, slope aspect; E4, slope gradient; E5, road quality; E6, distance between population and road; E7, surrounding population density; E8, land use type.

CCA showed that there was a significant correlation (*P* = 0.002) between the eight variables and the ordination axes, using the Monte Carlo permutation test. The appearance frequencies of all species were affected by the eight factors, with a decreasing order of importance: altitude, land use type, distance between population and road, road quality, slope aspect, surrounding population density, canopy density, and slope gradient (Figure 2A). The most important factor affecting the appearance frequency of orchid species was altitude (Figure 2B), while the most important factor affecting the appearance frequency of non-orchid species was distance between population and road (Figure 2C).

Among the orchids, six species with less than 5 distribution sites and 100 individuals (see Appendix S1) were significantly and positively correlated with altitude (*P*<0.05), but were significantly negatively correlated with surrounding population density and agricultural activities (*P*<0.01). Three species with less than 5 distribution sites and more than 100 individuals (see Appendix S1) had significant positive correlations with altitude and distance between population and road (*P*<0.05). Three species with more than 5 distribution sites and 100 individuals (see Appendix S1) had significant negative correlations with altitude, canopy density, and distance between population and road (*P*<0.05), but were significantly and positively correlated with agricultural activities (*P*<0.05) (Table 2).

**Table 2:** Bivariate correlation analysis between the appearance of the extremely small populations and the measured variables.

Group	Number of distribution sites	Number of individuals	Species	Significant correlation with effecting factors
Orchid species	≤5	≤100	12, 13, 14, 15, 16, 17	E2(0.619*), E7 (−0.790**), E8 (−0.760**)
	≤5	>100	11, 18, 20	E2 (0.607*), E6 (0.515*)
	>5	>100	9, 10, 19	E2 (−0.516*), E1 (−0.575*), E6 (−0.578*), E8 (0.510*)
Non-Orchid species	≤5	≤100	2, 3, 6, 7, 8	E8 (−0.950**), E5 (−0.754**), E6 (0.574*)
	≤5	>100	1	E5 (−0.612*), E7 (−0.456*)
	>5	≤100	5	E6 (0.848**), E7 (−0.602*), E8 (−0.738**)
	>5	>100	4	E2 (0.615*), E6 (0.698*), E8 (−0.753**)

* Significant at the 5% level of probability, ** Significant at the 1% level of probability.

The numbers from 1 to 20 represent the 20 species with extremely small populations, as shown in Appendix S1. E1, canopy density; E2, altitude; E3, slope aspect; E5, road quality; E6, distance between population and road; E7, surrounding population density; E8, land use type.

For non-orchid species, five species with less than 5 distribution sites and 100 individuals (see Appendix S1) had significant negative correlations with road quality and agricultural activities (*P*<0.01), but were significantly positively correlated with distance between population and road (*P*<0.05). One species with less than 5 distribution sites and more than 100 individuals (see Appendix S1) had significant negative correlations with road quality and surrounding population density (*P*<0.05). Two species with more than 5 distribution sites had significant positive correlations with altitude, and distance between population and road (*P*<0.05), but had extremely significant negative correlations with agricultural activities (*P*<0.01) (Table 2).

### Relationships Between Species Richness and Affecting Factors

**Figure 3:**
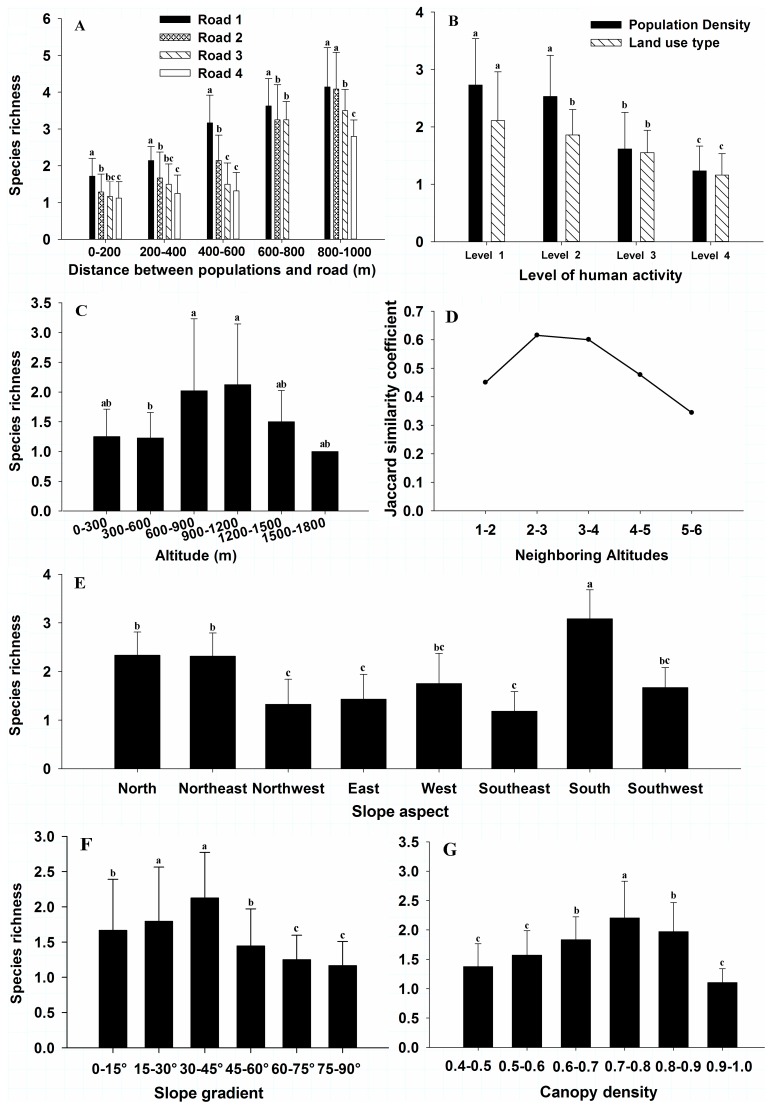
The relationship between eight factors and the species richness of extremely small populations (mean and standard errors). (A), distance between populations and road (Road 1, Road 2, Road 3, and Road 4 represent rural sandstone road, rural cement road, township road, and county road, respectively); (B), different levels of surrounding population density and land use; (C), different levels of altitudes; (D), coefficient similarity values of neighboring altitudes (the numbers from 1 to 6 represent altitude ranges of 0–300 m, 300–600 m, 600–900 m, 900–1200 m, 1200–1500 m, and 1500–1800 m); (E), different levels of slope aspects; (F), different levels of slope gradients; (G), different levels of canopy density. Different Lowercase letters (a, b, c) indicate significant difference at p<0.05.

The species richness of plant species with extremely small populations decreased with the increasing levels of road quality, agricultural activities, and surrounding population density, but increased with increasing distance of population and road (Figure 3A, 3B). The species richness differed significantly among the five levels of distance between populations and road, as well as the four levels of road quality (Distance: *F*_(4, 103)_ = 43.43, *P*<0.001; Road Quality: *F*_(3, 104)_ = 5.86, *P* = 0.001; Figure 3A), with largest distance of 800–1000 m, and a rural sandstone road having the peak richness of species. The species richness among the four levels of population density or agricultural activities were significantly different (Population Density: *F*_(3, 104)_ = 32.55, *P*<0.001; Agricultural Activities: *F*_(3, 104)_ = 12.22, *P*<0.001; Figure 3B), with a depopulated zone or dense forest having the peak richness of species.

The species richness of extremely small populations showed a unimodal curve with the increasing altitude. The species richness among six levels of altitudes were significantly different (*F*_(5, 102)_ = 3.98, *P* = 0.002; Figure 3C), with medium altitudes of 900–1200 m having the peak richness of species. The largest Jaccard similarity coefficient values for the extremely small populations were observed between two neighboring altitudes, including 600–900 m and 900–1200 m, and 900–1200 m and 1200–1500 m (Figure 3D).

The species richness among the eight levels of slope aspects were significantly different (*F*_(7, 100)_ = 2.16, *P* = 0.012; Figure 3E), with south aspect having the peak richness of species. Two species (*Chieniodendron hainanense, Dendrobium hainanense*) were found on all slope aspects. Three species (i.e. *Horsfieldia kingii, Paranephelium hainanense, Gastrochilus acinacifolius*) were mainly observed on the north, northeast, and northwest aspects. Seven species (i.e. *Cycas changjiangensis, Thrixspermum odoratum, Doritis pulcherrima, Dendrobium strongylanthum, Cymbidium eburneum, Pinalia quinquelamellosa, Dendrolirium tomentosum*) mainly occurred on the south, southeast, and southwest aspects (Table 3).

**Table 3:** Frequency of extremely small populations appearing on different slope aspects.

Species	North	Northeast	Northwest	East	West	Southeast	South	Southwest
1	0	0	0	0	0	0	0.75	0.25
2	0	0.5	0	0	0	0	0.125	0.375
3	0.444	0.333	0.111	0	0	0.111	0	0
4	0.154	0.076	0	0.154	0.076	0.385	0.076	0.076
5	0.333	0.083	0.167	0.083	0.083	0.083	0.083	0.083
6	0.250	0.375	0	0.125	0.250	0	0	0
7	0	0	0	0	0	0	1.000	0
8	0	0	0	0	0	0	1.000	0
9	0.079	0.105	0.079	0.105	0.105	0.184	0.211	0.132
10	0.222	0.222	0.111	0	0.278	0	0.056	0.111
11	0.182	0.045	0.273	0	0.318	0	0.091	0.091
12	0	0	0	0	0	0.667	0	0.333
13	0	0	0	0.167	0	0	0.167	0.667
14	0.400	0.200	0.400	0	0	0	0	0
15	0	0	0	0	0	0	1.000	0
16	0	0	0	0	0	1.000	0	0
17	0	0	0	0.500	0.500	0	0	0
18	0	0	0	0	0	0.333	0.333	0.333
19	0	0	0.111	0	0	0.333	0.333	0.222
20	0	0.125	0	0.125	0.125	0	0.375	0.250

The numbers from 1 to 20 represent the 20 species with extremely small populations, as shown in Appendix S1.

The species richness among the six levels of slope gradient were significantly different (*F*_(5, 102)_ = 3.99, *P* = 0.002; Figure 3F), with the medium level of slope gradient range from 30° to 45° having the peak richness of species. The slope gradient range from 30° to 45° had a larger Pearson similarity coefficient values with the ranges of 0°–15°, 15°–30°, 45°–60°, and 60–75° respectively, than those between other slope gradient (Table 4).

**Table 4:** Distance correlation analysis between extremely small populations and different slope gradients.

	1	2	3	4	5	6
**1**	1.000					
**2**	0.079	1.000				
**3**	0.663	0.426	1.000			
**4**	0.025	0.065	0.583	1.000		
**5**	0.209	0.161	0.412	0.396	1.000	
**6**	0.478	0.152	0.189	0.122	0.357	1.000

The numbers from 1 to 6 represent slope gradient ranges of 0°–15°, 15°–30°, 30°–45°, 45°–60°, 60°–75°, and 75°–90°.

The species richness among the six levels of canopy density were significantly different (*F*_(5, 102)_ = 13.34, *P*<0.001; Figure 3G), with the medium level of canopy density range 0.7–0.8 having the peak richness of species. The canopy density ranged from 0.7 to 0.8 had a larger Pearson similarity coefficient values with the ranges of 0.4–0.5, 0.5–0.6, 0.6–0.7, and 0.8–0.9 respectively, than those between other canopy density (Table 5).

**Table 5:** Distance correlation analysis between extremely small populations and different canopy densities.

	1	2	3	4	5	6
**1**	1.000					
**2**	0.454	1.000				
**3**	0.243	0.000	1.000			
**4**	0.375	0.564	0.342	1.000		
**5**	0.193	0.152	0.124	0.344	1.000	
**6**	0.059	0.130	0.243	0.193	0.150	1.000

The numbers from 1 to 6 represent canopy density ranges of 0.4–0.5, 0.5–0.6, 0.6–0.7, 0.7–0.8, 0.8–0.9, and 0.9–1.0.

## Discussion

### Factors Affecting the Appearance Frequency

As expected, we found that plant species with extremely small populations often lived in a fragmental habitat (Appendix S1). For example, four species (i.e. *Ilex kaushue*, *Bulbophyllum hainanense*, *Phaius hainanensis*, *Sunipia hainanensis*) with extremely small populations were not found in our field survey, and the remaining 20 species underwent human disturbances and threats. Thus the population size of these species are smaller than the minimum number requirements inhibiting from extinction, suggesting that these extremely small populations face the threat of extinction if no protective actions are taken. Our findings have been demonstrated by the view that the main factors causing rare and endangered plants susceptible to extinction are habitat destruction, plant overexploitation and the ecological environment deterioration [25], [35], [36].

Although there is one or several factors causing plant species endangerment, these factors may be completely different for different species [35]. Therefore, we found that the leading factors changing the appearance frequency of extremely small populations differ among plant species. For example, the dominant factors affecting the appearance frequency of orchid and non-orchid species were altitude and the distance between population and road, respectively (Figure 2B, Figure 2C).

For most of the endangered plants, the direct factors leading them susceptible to extinction are human activities [25], [36], [37]. This point is proved by our results that the appearance frequencies of 17 species with extremely small populations had significant negative correlations with road quality, agricultural activities, or the surrounding human population density (Table 2). These findings may result from that most of these species have a highly economic utilization value or excellent ornamental characteristics, and thus that they are often overexploited by local people.

In this study, most orchid species were concentrated in high altitudinal habitats (Table 2). Seed germination and growth of some orchids are dependent on high altitudinal habitats, which not only have favorable temperatures and higher humidity, but also avoid human disturbance [38], [39]. Additionally, we found that the appearance frequencies of three orchid species (*Dendrobium hainanense, Oxystophyllum changjiangense, Dendrolirium tomentosum*) were significantly and positively correlated with roads and agricultural activities (Table 2). These species were also concentrated along roadsides and in areas of human activity (Appendix S1), suggesting that the distribution of some endangered orchid species benefit from human activities or roads. Roads usually have larger negative effects on the distribution of native plants than positive effects [40], [41]. However, the frequent appearance of the orchid species in this study may probably result from that the survival of these species benefits from road disturbance [42], and that an intermediate level of disturbance can help prevent them from becoming endangered [43].

### Factors Affecting Species Richness

Our results showed that the species richness of extremely small populations was always reduced by human activities (Figure 3A, 3B). The main factor causing the decreasing species richness for these species probably being the habitat destruction and fragment because of human disturbance, such as land use type, roads, etc [16], [44], [45], [46]. For example, we found that road quality made a decreasing richness of the extremely small populations.

In this study we found that the peak richness of the small populations always occurred at the medium level across an environmental gradient (Figure 3C, 3F, and 3G), and it also had a larger species similarity values (Figure 3D, Table 4, Table 5). Our results were confirmed by many previous studies [47], [48], [49], [50]. For example, Jiang et al. (2002) and Cui et al. (2006) found that many rare and endangered plants are often concentrated at medium altitudes [51], [52]. On the other hand, slope gradient can reflect the soil moisture and its nutrient condition [53], [54], the distribution of most rare plants are concentrated in a medium level of slope gradients with favorable soil conditions [55], [56]. Also, since the increasing forest canopy can improve the forest microhabitat conditions, and regulate the distribution, composition, and growth of forest subordinate layers [57], the seedlings of most of the rare and endangered plants thus grew better with high abundance under medium canopy densities [58].

Our results showed that the peak richness of extremely small populations always appeared at the south aspect, and that their frequencies were always the largest on sunny or semi-sunny slope aspects (Figure 3E, Table 4), due to their drought tolerance and sun preference. But several other studies have shown that the species richness of endangered plants and their individual numbers is higher on shady and semi-shady slope aspects than those on other aspects, because they prefer to high humidity and low irradiance [55], [59], [60]. These inconsistent results may account for that the different extremely small populations can adapt to different habitats with different humidity and irradiance. We, therefore, should take knowledge of how the external factors, such as the ecological environment, land use type, roads, human activity, etc., affect the distribution of the extremely small populations for the better protecting them in the future.

## Supporting Information

Appendix S1 **Status of extremely small populations in Hainan.**
(DOC)
