# Supplementary material for: Factors Affecting the Distribution Pattern of Wild Plants with Extremely Small Populations in Hainan Island, China
Source: PLoS One. 2014 May 15;9(5):e97751. doi: 10.1371/journal.pone.0097751 (PMC4022659; doi:10.1371/journal.pone.0097751)
Supplement: Appendix S1 — Status of extremely small populations in Hainan. (DOC) [file pone.0097751.s001.doc]

**Factors affecting the distribution pattern of wild plants with extremely small populations in Hainan Island, China**

Yukai Chen^1^, Xiaobo Yang^1^*, Qi Yang^1^, Donghai Li^1^, Wenxing Long^1^, Wenqi Luo^1^

1Key Laboratory of Protection and Development Utilization of Tropical Crop Germplasm Resources, Ministry of Education; College of Horticulture and Landscapes, Hainan University, Haikou 570228, Hainan, China.

* Author for correspondence. Email: [yanfengxb@163.com](mailto:yanfengxb@163.com). Tel.: 86898-66279131; Fax: 86898-66180571.

**Appendix S1. Status of extremely small populations in Hainan.** The numbers from 1 to 20 in the first row represent the extremely small population species of the study area: 1, *Cycas changjiangensis*; 2, *Michelia odora*; 3, *Horsfieldia kingii*; 4, *Hopea hainanensis*; 5, *Chieniodendron hainanense*; 6, *Paranephelium hainanense*; 7, *Sonneratia hainanensis*; 8, *Lumnitzera littorea*; 9, *Dendrobium hainanense*; 10, *Oxystophyllum changjiangense*; 11, *Dendrobium sinense*; 12, *Dendrobium strongylanthum*; 13, *Thrixspermum odoratum*; 14, *Gastrochilus acinacifolius*; 15, *Doritis pulcherrima*; 16, *Cymbidium eburneum*; 17, *Cymbidium insigne*; 18, *Pinalia quinquelamellosa*; 19, *Dendrolirium tomentosum*; 20, *Ceratostylis hainanensis*. CR, critically endangered; EN, endangered; NT, near threatened; VU, vulnerable; ENR, distributed entirely in the National Nature Reserve; PNR, partly distributed in the national reserve; EPR, distributed entirely in the Provincial Nature Reserve; ONR, distributed entirely outside of reserve areas.

| Specie | IUCN  red list | Endemic | Distribution area | Number of distribution sites | Number of individuals | Habitat |
| --- | --- | --- | --- | --- | --- | --- |
| 1 | CR | Endemic | ENR | 1 | 320 | under forest of *Pinus latteri*, forest margin |
| 2 | NT | - | ENR | 1 | 7 | forest margin |
| 3 | - | - | PNR | 5 | 13 | road side, forest margin |
| 4 | CR | - | PNR | 12 | 155 | in the plantation, road side |
| 5 | VU | - | PNR | 6 | 40 | road side, forest margin |
| 6 | - | Endemic | ONR | 1 | 30 | road side, forest margin |
| 7 | CR | Endemic | EPR | 1 | 3 | near the village |
| 8 | - | - | ONR | 2 | 40 | near the village |
| 9 | - | Endemic | PNR | 19 | 800 | in the plantation, road side, forest margin |
| 10 | EN | Endemic | PNR | 12 | 200 | in the plantation, road side, forest margin |
| 11 | EN | Endemic | ENR | 5 | 300 | tree trunk of high-altitude |
| 12 | - | - | ENR | 1 | 20 | forest margin of high-altitude |
| 13 | - | - | PNR | 3 | 30 | forest margin of high-altitude |
| 14 | - | Endemic | ONR | 1 | 50 | tree trunk of high-altitude |
| 15 | - | - | PNR | 2 | 50 | rock of roadside |
| 16 | - | - | PNR | 2 | 5 | road side, tree trunk of high-altitude |
| 17 | - | - | PNR | 3 | 50 | [mountain ridge](javascript:showjdsw('showlj_1','lj_1')) of high-altitude |
| 18 | - | Endemic | ENR | 1 | 110 | tree trunk of high-altitude |
| 19 | - | - | PNR | 8 | 110 | in the plantation, road side |
| 20 | - | Endemic | PNR | 5 | 200 | tree trunk of high-altitude |
